# Solitary pulmonary capillary hemangioma presenting with a ground glass opacity: A case report & literature review

**DOI:** 10.1016/j.ijscr.2020.08.020

**Published:** 2020-08-29

**Authors:** Teruya Komatsu, Akira Hara, Naoki Date, Takuji Fujinaga, Tatsuo Kato

**Affiliations:** a Department of General Thoracic Surgery, National Hospital Organization Nagara Medical Center, Gifu, Japan; b Department of Tumor Pathology, Gifu University Graduate School of Medicine, Gifu, Japan; c Department of Respiratory Medicine, National Hospital Organization Nagara Medical Center, Gifu, Japan

**Keywords:** Benign lung tumor, Solitary pulmonary capillary hemangioma, Ground glass opacity, Video-assisted thoracoscopic surgery, Case report

## Abstract

**Introduction:**

Solitary pulmonary capillary hemangioma (SPCH) is a rare benign lung tumor that clinically resembles early lung cancer and precancerous pulmonary lesions that present with similar imaging manifestations.

**Presentation of case:**

The patient was a 54-year-old Japanese man who was referred to Nagara Medical Center with a ground glass opacity (GGO) lesion within the right upper lung that was incidentally detected on computed tomography. After 8 months of follow-up, video-assisted thoracoscopic segmental resection of the right upper lobe was performed with diagnostic and therapeutic intent. Pathologic examination of the resected specimen demonstrated thickening of the alveolar septum caused by the proliferation of capillary vessels. This lesion was positive for CD31 and CD34 and negative for thyroid transcription factor-1 and cytokeratin on immunohistochemical staining. The tumor was diagnosed as SPCH pathologically.

**Discussion:**

When radiological examination demonstrates a GGO in the lung, SPCH must be considered as one of the differential diagnoses. For a definitive diagnosis, pathological examination of a surgically resected specimen must be conducted.

**Conclusion:**

This study describes a case of SPCH and a review of the literature.

## Highlights

• Solitary pulmonary capillary hemangioma (SPCH) is a rare benign lung tumor.
• SPCH presents with a ground glass opacity on CT scan.
• Preoperative definitive diagnosis as SPCH is a real challenge.
• Immunohistochemical staining is essential for diagnosis as SPCH.

## 1 Introduction

Solitary pulmonary capillary hemangioma (SPCH) is a rare benign lung tumor [1]. Preoperative diagnosis remains a challenge because it is radiographically visualized as a ground glass opacity (GGO), which is considered to indicate early lung cancer or a precancerous lesion [1,2]. Definitive diagnosis as SPCH needs immunohistochemical staining [2,3]. We report a case of SPCH and a review of the literature.

This case is reported in line with the SCARE criteria [4].

## 2 Presentation of case

**Fig. 1:**
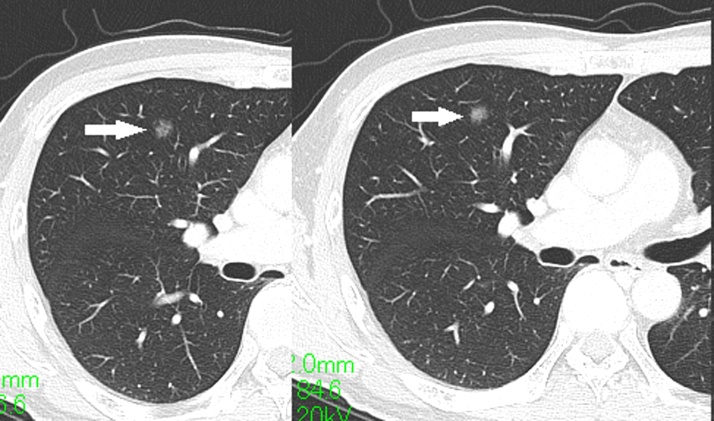
Computed tomography (CT) manifestation of the lung lesion (arrowheads). Chest CT showing a pure ground glass opacity, with a maximum diameter of 8 mm, located in the subpleural area of the right upper lung.

**Fig. 2:**
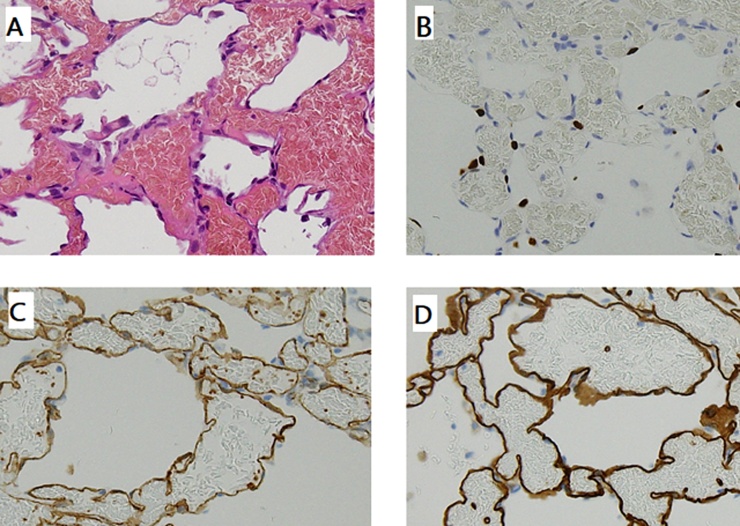
(A) Histopathologic features of a solitary pulmonary capillary hemangioma (SPCH). Hematoxylin and eosin staining (original magnification, ×200) show alveolar structures with proliferated capillaries and enlarged endothelial cells without cytological atypia. (B–D) Immunohistochemical staining of the SPCH (original magnification, ×200) revealed positivity for thyroid transcription factor-1 (B) in the nuclei of alveolar epithelial cells and for CD31 (C) and CD34 (D) in the cytoplasm of endothelial cells.

A 54-year-old Japanese man was referred to Nagara Medical Center for the evaluation of a GGO, which was found incidentally within the right upper lung on computed tomography (CT). Chest CT showed a pure GGO lesion measuring 8 mm in diameter in the anterior segment of the right upper lobe (Fig. 1). The patient had quit smoking approximately 15 years previously. He had regularly seen a family doctor for chronic gastritis. The laboratory workup, including tests for tumor markers such as carcinoembryonic antigen, squamous cell carcinoma antigen, and cytokeratin 19 fragment, did not show remarkable results. The lesion was suspected to be a slow-growing early-stage non-small cell lung cancer. For diagnostic and therapeutic purposes, a segmentectomy (anterior segment of the right upper lobe) was performed via video-assisted thoracoscopic surgery. Pathologic examination showed thickening of the alveolar septum caused by the proliferation of capillary vessels without cytological atypia (Fig. 2A). Immunohistochemistry revealed that this lesion was negative for thyroid transcription factor-1 (TTF-1, Fig. 2B) and cytokeratin and positive for CD31 and CD34 (Fig. 2C and D). The final diagnosis was SPCH. The patient’s postoperative course was uneventful.

## 3 Discussion

The ﬁrst report of a surgically resected case of SPCH was published by Fugo et al. in 2006 [5]. The number of reported cases has increased to 33 in the English literature; however, it is still a rare benign lung tumor accounting for only 8.5% of all benign lung nodules [1].

SPCH is difficult to diagnose because of a lack of typical symptoms. Radiographic findings often include pure or mixed GGOs, which may lead to suspicions of adenocarcinoma in situ (AIS), atypical adenomatous hyperplasia, and focal inflammation [2,6]. In their case series, Zhao et al. summarized 9 surgically resected cases of SPCH. Approximately 30% of all patients were men aged 37–63 (median 47) years. The size of the tumors ranged from 6 to 25 (median 13) mm. In terms of radiographic appearance, the following findings were observed on chest CT: 3 cases, pure GGO; 3 cases, mixed GGO; 2 cases, a completely solid nodule; and 1 case, a cystic-solid appearance [2]. According to the report by Sakaguchi et al., 18F-fluorodeoxyglucose positron emission tomography for SPCH showed no abnormal uptake in the lesion [7]. Therefore, discriminating SPCH from these differential diagnoses based on imaging findings alone is challenging [8,9].

Microscopically, an SPCH typically appears as a solitary lesion with densely proliferating and dilated capillaries within the alveolar septum, which is composed of a single layer of ﬂattened or cuboidal endothelial cells without cytological atypia [2,3]. The increased capillary density caused by hyperplasia and the enlarged endothelial cells lead to the appearance of a mixed GGO rather than a pure GGO on chest CT [2].

The characteristic immunohistochemical findings of SPCH have been reported. The expression of epithelial cell and histiocyte markers, such as cytokeratin, TTF-1, and CD68 (KP-1), is negative. Cells of the alveolar septa are positive for CD31 and CD34 [2,3].

All reported patients with SPCH were asymptomatic. No patients have died from the disease [3]. However, the frequency of the diagnosis of SPCH is likely to increase with the widespread use of CT for lung cancer screening [1]. Preoperative differential diagnosis based on imaging findings is very difficult. Intraoperative frozen section analysis poses a challenge with respect to intraoperative decision making [10].

## 4 Conclusion

GGO lesions may represent an SPCH or AIS/early lung cancer. Because the prognosis of each of these diseases is quite different, a pathological examination involving immunohistochemistry must be conducted to ensure the establishment of the correct diagnosis.

## Conflicts of interest

The authors declare that they have no competing interests.

## Sources of funding

No sources of funding.

## Ethical approval

We have reported a single case, not a clinical study, with no requirement for ethical approval.

## Consent

Written informed consent was obtained from the patient for publication of this case report and accompanying images. A copy of the written consent is available for review by the Editor-in-Chief of this journal on request.

## Author contribution

Dr Teruya Komatsu: Investigation, Writing – original draft, Writing – Review and Editing, Conceptualization, Visualisation.

Dr Naoki Date and Dr Takuji Fujinaga: Conceptualization, Writing – Review and Editing.

Dr Akira Hara and Dr Tatsuo Kato: Writing – Review and Editing, Supervision.

## Registration of research studies

Not applicable.

## Guarantor

Teruya Komatsu and Takuji Fujinaga are the guarantors of this work. Thus, they have full access to all study data and take responsibility for the integrity and accuracy of such data.

## Provenance and peer review

Not commissioned, externally peer-reviewed.
